# PCIPG2.0: multi-omics fusion and structure-aware graph autoencoding for protein complex identification

**DOI:** 10.1093/bioinformatics/btag552

**Published:** 2026-07-25

**Authors:** Yixiang Huang, Jiudong Wang, Lei Yang, Xinqi Gong

**Affiliations:** School of Mathematics, Renmin University of China, Beijing 100872, China; School of Life Science, Beijing Institute of Technology, Beijing 100081, China; School of Mathematics, Renmin University of China, Beijing 100872, China; School of Mathematics, Renmin University of China, Beijing 100872, China

## Abstract

**Motivation:**

Protein complexes execute cellular functions, yet identifying them from protein–protein interaction (PPI) networks remains challenging because interactomes are incomplete and purely topology-driven clustering often lacks mechanistic interpretability. Here we present PCIPG 2.0, an unsupervised framework that explicitly addresses two major bottlenecks in PPI-based complex discovery: missing interactions and limited mechanistic specificity. PCIPG 2.0 first fuses multiple omics views to prioritize high-confidence candidate protein associations and enhance the observed interactome, and then learns structure-aware node representations by aggregating residue embeddings on residue graphs, coupled with a PPI-level graph autoencoder to infer latent complex memberships.

**Results:**

Across five yeast benchmarks, PCIPG 2.0 consistently improves complex recovery over representative baselines and yields predicted complexes with significantly higher Gene Ontology semantic coherence than size-matched random sets. Literature-supported case studies and AlphaFold3-based assembly analyses further suggest that representative predictions are consistent with functionally coherent and structurally plausible protein assemblies. Together, these results suggest that combining multi-omics-driven interactome completion with residue-informed representation learning provides a useful and mechanistically informed framework for protein complex identification under incomplete interactome measurements.

**Availability:**

The source code is available at GitHub: https://github.com/hyx-1/PCIPG2.0. The complete reproducibility package, including the code, processed data, configuration files and materials required to reproduce the experiments reported in this manuscript, has been archived on Zenodo with an archival DOI: https://doi.org/10.5281/zenodo.20133228. The GitHub repository also provides the README-based usage instructions.

## 1 Introduction

Protein–protein interactions (PPIs) underpin cooperative molecular processes in cells, and proteins frequently assemble into stable or dynamic complexes to execute biological functions, playing central roles in transcriptional regulation, signal transduction and metabolic control. Accordingly, robust identification of protein complexes from large-scale interaction maps is central to understanding cellular organization, dissecting disease mechanisms and prioritizing candidate drug targets. Advances in high-throughput assays such as yeast two-hybrid (Y2H) and affinity purification–mass spectrometry (AP–MS) have enabled interactome-scale PPI atlases across organisms (Ito *et al.* 2001, Gavin *et al.* 2006). However, currently available interactomes remain incomplete and noisy, with pervasive false negatives that obscure native assembly patterns. Methodological limitations—including reduced sensitivity to weak or transient interactions and dependence on experimental conditions—contribute to limited coverage and cross-study inconsistency (Peng *et al.* 2017), which in turn can impair network-based module detection and downstream functional interpretation.

Computational methods for protein complex identification have evolved along two main lines: unsupervised and supervised learning. Unsupervised approaches identify complexes by exploiting PPI topology, ranging from early dense-subgraph expansion and local scoring schemes such as MCODE (Bader and Hogue 2003) and LCMA (Li *et al.* 2005), to flow- and ensemble-based clustering (MCL (Asur *et al.* 2007)) and overlapping-community formulations (CFinder (Adamcsek *et al.* 2006) and ClusterONE (Nepusz *et al.* 2012)) that better accommodate proteins participating in multiple modules. Inspired by proteome-scale complex organization, the core–attachment view (Gavin *et al.* 2006) has been incorporated into complex identification via methods such as CORE (Leung *et al.* 2009) and COACH (Wu *et al.* 2009), and subsequent work has improved robustness by weighting interaction reliability or leveraging structural/topological similarity, for example EWCA (Wang *et al.* 2019a). With the rapid development of machine learning, heuristic designs have been complemented by representation learning approaches that integrate local and global topology, including network compression and DeepWalk-based embeddings (DPC-HCNE (Meng *et al.* 2019)), partition-driven coherent clustering (PC2P (Omranian *et al.* 2021)), and cohesion-aware spectral clustering (TADW-SC (Berahmand *et al.* 2021)). In parallel, supervised methods learn discriminative patterns from curated gold-standard complexes, including generative probabilistic formulations (SCI-BN (Qi *et al.* 2008)), embedding-plus-classifier pipelines (Node2vec-RF (Wang *et al.* 2022)), adversarial learning (PCGAN (Pan *et al.* 2023)), adaptive graph convolution integrating functional annotations (AdaPPI (Chen *et al.* 2023)), and hypergraph encoders that incorporate sequence features (HyperGraphComplex (Xia *et al.* 2024)). Beyond topology, functional knowledge bases have increasingly been used to augment PPI modeling and complex discovery: GO-driven attribute modeling with Markov graph networks (PCIA (Hu and Chan 2013)); GO-weighted similarity networks combined with cluster mining (GANE (Xu *et al.* 2018); SE-DMTG (Wang *et al.* 2019b)); transcriptome–PPI fusion in graph neural networks for link prediction–driven discovery (DeepSignalingLinkNet (Feng *et al.* 2020)); and dynamic PPI formulations from time-varying expression to capture condition-dependent modules (CPredictor4.0 (Shi *et al.* 2018); CO-DPC (Xiao *et al.* 2019)).

Despite this progress, two limitations remain. First, most methods treat unobserved protein pairs as non-interacting, without explicitly modeling missing edges, even though coverage and ascertainment bias can distort functional association signals and compromise clustering (Vidal *et al.* 2011, Schaefer *et al.* 2013). Although reweighting strategies and similarity-based compensation can partially mitigate noise, they often depend on a single data source and underutilize the complementarity of multi-omics evidence. Second, complex identification is frequently performed at the protein level, overlooking the residue- and interface-level physical determinants of PPIs. Direct interactions are mediated by a limited set of interface residues and their three-dimensional complementarity; structural studies show that physicochemical properties and spatial packing at interfaces are key determinants of complex stability and specificity (Bogan and Thorn 1998). Beyond interface-specific determinants, explicit geometric descriptions of protein structures have also been used for structural characterization and functional analysis. For example, amino-acid torsion angles have been used to predict protein fold classifications, whereas the three-dimensional Yau–Hausdorff distance has been applied to protein structure comparison and function inference (Tian *et al.* 2019, 2020). Although advances in structure prediction have greatly expanded structural coverage (Jumper *et al.* 2021), systematic integration of residue-level structural cues into interactome modeling and complex discovery remains limited.

**Figure 1:**
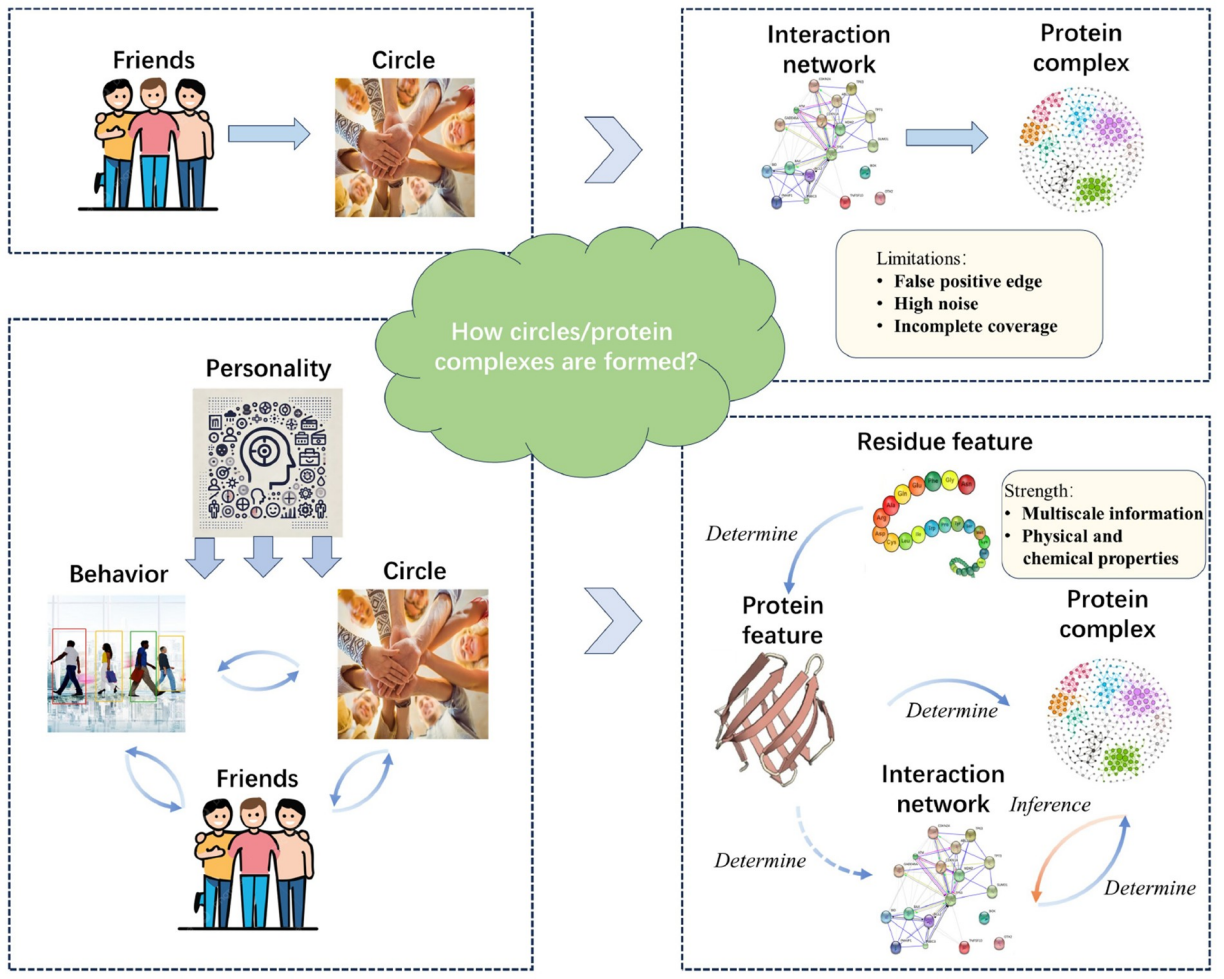
Multiscale rationale of PCIPG2.0 for protein complex discovery. Top, conventional complex identification treats the PPI network as the sole substrate for module detection, but is hindered by false-positive edges, high noise and incomplete coverage. Bottom, we use a simple social analogy as an intuition: residue physicochemical traits correspond to “personality,” protein structure reflects “behavior,” pairwise PPIs represent “friendships,” and protein complexes emerge as higher-order “circles.” Guided by this hierarchy, residue-level features are aggregated into structure-aware protein representations, which together with interaction evidence are integrated to infer complex membership, enabling complex discovery that is both mechanistically grounded and robust to noise and missing interactions.

To summarize these multi-scale dependencies, we can view complex assembly through a simple social analogy: residue-level determinants shape interaction compatibility, PPIs correspond to measurable pairwise relationships, and complexes emerge as higher-order groups stabilized by multiple reinforcing interactions. We use this analogy as an intuition. Methodologically, the central challenge is to combine missing-edge recovery and residue-informed representation learning within a unified framework for complex inference under incomplete and noisy interactome measurements (Fig. 1).

To address this gap, we present PCIPG 2.0, which extends PCIPG 1.0 by introducing multi-omics fusion–driven interactome completion as a first-class component of the framework. Its first stage uses complementary omics evidence to prioritize likely missing edges before complex inference, thereby mitigating false negatives that cannot be recovered from topology alone. Its second stage derives structure-aware protein representations by encoding residue neighborhoods with the ProteinMPNN encoder and propagating signals on residue contact graphs via graph convolution, followed by protein-level pooling. Finally, the completed interaction graph and structural node features are integrated within an unsupervised graph autoencoder (GIN encoder with an inner-product decoder), yielding latent complex-membership factors that support downstream complex extraction with calibrated confidence. In this way, PCIPG 2.0 combines multi-omics–driven interactome completion and residue-informed representation learning within a single unsupervised framework for protein complex discovery.

## 2 Materials and methods

### 2.1 Overview of PCIPG 2.0

**Figure 2:**
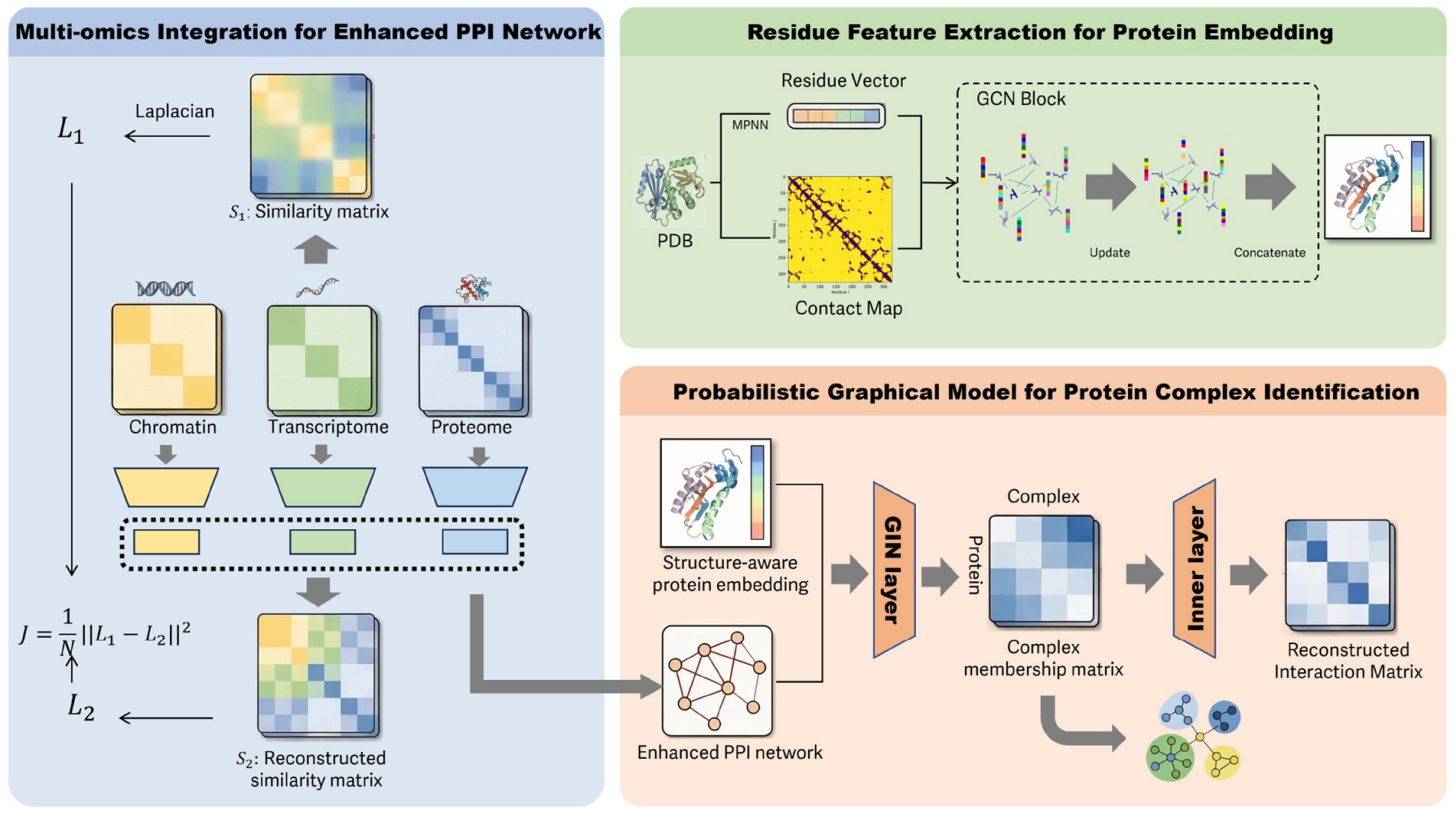
PCIPG2.0 workflow: multi-omics fusion-driven interactome completion with structure-aware graph autoencoding for protein complex inference. Left, PCIPG2.0 integrates complementary omics layers (chromatin, transcriptome and proteome) by building cross-view similarity graphs and learning view-specific mappings into a shared latent space. Multi-view structure consistency is enforced by aligning graph structures between the input space and the embedding space, producing a fused representation that prioritizes high-confidence candidate edges and yields an enhanced PPI network. Top right, residue-level structural information is extracted from PDB structures using the ProteinMPNN encoder and propagated on residue contact maps with GCN blocks to obtain structure-aware protein embeddings. Bottom right, the enhanced interaction graph and structure-informed node features are integrated within an unsupervised graph autoencoder (GIN encoder with inner-product decoding) to learn a complex membership matrix and reconstruct interaction patterns, enabling downstream extraction of protein complexes from the inferred latent factors.

PCIPG 2.0 is an unsupervised framework for protein complex identification that couples multi-omics evidence integration with structure-aware representation learning on PPI networks (Fig. 2). The method proceeds in two stages. First, a multi-omics fusion module aligns multiple omics views into a shared embedding space and uses this representation to prioritize high-confidence protein pairs, thereby augmenting the observed interactome with candidate edges to reduce the impact of missing interactions. Second, structural information is incorporated by extracting residue-level embeddings from protein structures, aggregating them into protein-level features, and using these features as node attributes for a PPI-level graph autoencoder. The autoencoder learns global protein embeddings and a latent complex-membership matrix from which discrete complexes are derived. Together, these components enable PCIPG 2.0 to exploit complementary omics signals and structural cues while retaining the ability to operate without complex labels.

### 2.2 Datasets and preprocessing

We evaluated PCIPG on *Saccharomyces cerevisiae*. The input PPI networks were obtained from five widely used yeast benchmarks: Collins, Krogan-core, Krogan14k, DIP and BioGRID (Table 1). Each network was modeled as an undirected graph, with proteins as nodes and physical interactions as edges. These datasets vary substantially in size, density and experimental origin, providing complementary regimes of sparsity and noise.

**Table 1:** Statistics of PPI datasets.

Dataset	Protein nodes	Interaction edges
Collins Collins *et al.* (2007)	1622	9074
Krogan-core Krogan *et al.* (2006)	2708	7123
Krogan14k Krogan *et al.* (2006)	3581	14 076
DIP Xenarios *et al.* (2002)	4928	17 201
BioGRID Oughtred *et al.* (2019)	5640	59 748

For quantitative evaluation, we assembled a gold-standard complex set following prior work (for example, AdaPPI). We integrated authoritative yeast complex annotations from MIPS (Mewes *et al.* 2006), CYC2008 (Pu *et al.* 2009), SGD (Wong *et al.* 2019), Aloy (Aloy *et al.* 2004) and TAP06 (Gavin *et al.* 2006), removed highly overlapping duplicates, and filtered out complexes with fewer than three proteins. The final reference set contains 789 complexes.

For multi-omics features, we incorporated transcriptomic and chromatin-related profiles from GEO (GSE168699) to provide functional context complementary to PPIs. Raw features from different omics views were mapped to yeast proteins under a unified identifier system and standardized independently before fusion. Proteins without unambiguous mapping in a given omics layer were excluded from that view during feature construction. When multiple features mapped to the same protein, we used max aggregation to obtain a unique protein-level feature vector. Missing values were handled using kNN imputation, followed by view-wise *z*-score normalization. Multi-omics information was used only to prioritize candidate missing edges for PPI network enhancement. Proteins without matched multi-omics features were retained in the original PPI network with their observed interactions unchanged, so the enhancement step did not alter the node set of the network.

For structural cues, we used the encoder component of ProteinMPNN (Dauparas *et al.* 2022) to extract residue-level embeddings from standardized protein structures. Each protein was represented as a residue graph with spatial adjacency, and the ProteinMPNN encoder outputs were used as initial residue features. Whenever experimentally resolved structures were available, we prioritized those structures for downstream graph construction. For proteins without experimentally resolved PDB structures, we used predicted models to obtain the corresponding structural coordinates. All structures were converted into a unified residue-level representation before graph construction, so that experimentally resolved and predicted structures were processed using the same downstream pipeline. In the final dataset, experimentally resolved structures were available for 58% of benchmark proteins, whereas the remaining proteins were assigned predicted models.

### 2.3 Multi-omics fusion and PPI network enhancement

The multi-omics fusion module aligns multiple omics views by enforcing consistency between the Laplacian structure in the input space and that in the learned embedding space, enabling effective cross-view integration and PPI network enhancement. Let $V=\{v_{1},\ldots ,v_{N}\}$ denote *N* proteins. Each protein is observed in *K* omics views. In view *k*, the input feature of protein *i* is $X_{i}^{k}$.

#### 2.3.1 Input-space similarity

We define the input-space similarity between protein *i* in view *k* and protein *j* in view *l* as


(1) $$S_{ij}^{kl}=\text{exp}\;\left(-\frac{d{\left(X_{i}^{k},X_{j}^{l}\right)}^{2}}{t}\right),$$

Where *t* is a temperature parameter and d(·,·) is the Euclidean distance. $S_{1}$ is a block matrix collecting all $\left\{S^{kl}\right\}$:


(2) $$\begin{array}{c} S_{1}=\left(\begin{array}{ccc} S^{11} & \cdots & S^{1K}\\ \vdots & \ddots & \vdots \\ S^{K1} & \cdots & S^{KK} \end{array}\right). \end{array}$$

#### 2.3.2 View-specific mappings and shared embeddings

For each view *k*, we learn a nonlinear mapping (implemented by an MLP) $f_{k}(\cdot ;\Theta_{k})$ to obtain view-specific embeddings:


(3) $$y_{i}^{k}=f_{k}(X_{i}^{k};\Theta_{k}),\;k=1,2,\ldots ,K$$

And define the fused embedding as


(4) $$Y_{i}=(y_{i}^{1},y_{i}^{2},\ldots ,y_{i}^{K}).$$

#### 2.3.3 Embedding-space similarity

We similarly define the embedding-space similarity as


(5) $${\hat{S}}_{ij}^{kl}=\text{exp}\;\left(-\frac{d{\left(y_{i}^{k},y_{j}^{l}\right)}^{2}}{\tau }\right),$$

Where τ is a temperature parameter. $S_{2}$ is the corresponding block matrix collecting all $\left\{{\hat{S}}^{kl}\right\}$:


(6) $$\begin{array}{c} S_{2}=\left(\begin{array}{ccc} {\hat{S}}^{11} & \cdots & {\hat{S}}^{1K}\\ \vdots & \ddots & \vdots \\ {\hat{S}}^{K1} & \cdots & {\hat{S}}^{KK} \end{array}\right). \end{array}$$

#### 2.3.4 Normalized Laplacians

Given a similarity matrix S, let $D_{ii}=\sum_{j}S_{ij}$ and define the random-walk normalized Laplacian as


(7) $$L=I-D^{-1}S.$$

We compute $L_{1}$ from $S_{1}$ and $L_{2}$ from $S_{2}$.

#### 2.3.5 Structure-consistency objective

We enforce structure consistency by minimizing


(8) $$L=\frac{1}{N}{\left\|L_{1}-L_{2}\right\|}_{F}^{2},$$

Where $||\cdot |{|}_{F}$ denotes the Frobenius norm.

#### 2.3.6 PPI network enhancement

After training, we compute Euclidean distances between proteins in the fused embedding space from Eq. 4, rank unobserved protein pairs, and add the top-*P* closest pairs as candidate edges to construct the enhanced PPI network adjacency matrix *A* (P=200):


(9) $$A_{ij}=\{\begin{array}{cc} 1, & \text{if}\;(i,j)\in \text{enhanced}\;\text{PPI}\;\text{network},\\ 0, & \text{if}\;(i,j)\notin \text{enhanced}\;\text{PPI}\;\text{network}. \end{array}$$

### 2.4 Structure-aware protein representation via residue-level graph aggregation

For each protein, we construct a residue-level graph representation based on its three-dimensional structure $G_{b}=\left(V_{b},E_{b},X_{b}\right)$, where $V_{b}$ denotes the set of residue nodes with $|V_{b}|=n$, $E_{b}$ encodes spatial adjacency between residues, and $X_{b}\in R^{n\times d}$ is the residue feature matrix whose *i*-th row corresponds to the initial representation of residue *i*. Specifically, we add an undirected edge $(i,j)\in E_{b}$ if the Cα–Cα distance satisfies d(i,j)≤8 Å.

#### 2.4.1 Residue-level GCN updates

We apply an *L*-layer graph convolutional network (GCN) on $G_{b}$. Let $H^{\left(l\right)}\in R^{n\times d_{l}}$ denote the residue representations at layer *l* (with $H^{\left(0\right)}=X_{b},d_{0}=d$). The propagation rule is


(10) $$H^{\left(l+1\right)}=\sigma \left({\tilde{D}}^{-\frac{1}{2}}\tilde{E}{\tilde{D}}^{-\frac{1}{2}}H^{\left(l\right)}W^{\left(l\right)}\right),$$

Where $\tilde{E}=E_{b}+I$ augments the residue adjacency with self-loops, $\tilde{D}$ is the corresponding degree matrix with ${\tilde{D}}_{ii}=\sum_{j}{\tilde{E}}_{ij}$, $W^{\left(l\right)}$ are learnable weight matrices, and σ(·) denotes the ReLU activation.

#### 2.4.2 Residue-to-protein pooling

Given the final-layer residue embeddings $H^{\left(L\right)}={\left\{h_{i}^{\left(L\right)}\right\}}_{i=1}^{n}$, we obtain a protein-level representation by global mean pooling:


(11) $$x_{p}=\frac{1}{n}\sum_{i=1}^{n}h_{i}^{\left(L\right)},\;p=1,2,\ldots ,N.$$

This module captures intra-protein structural dependencies. The resulting protein embeddings $x_{p}$ are used as node features for subsequent PPI-level learning.

### 2.5 PPI-level graph autoencoder for complex prediction

We constructed an enhanced PPI graph $G_{t}=(V_{t},E_{t},X_{t})$ with *N* proteins, where an undirected edge $(i,j)\in E_{t}$ if $A_{ij}=1$. Given $G_{t}$, we train a graph autoencoder to learn protein representations without using complex labels. Node features $X_{t}\in R^{N\times c_{t}}$ are provided by the structure-aware protein module.

#### 2.5.1 GIN encoder

We stack three graph isomorphism network (GIN) layers. Let $Z_{i}^{\left(l\right)}$ denote the representation of node *i* at layer *l* with $Z_{i}^{\left(0\right)}=x_{i}$. The update is


(12) $$Z_{i}^{\left(l+1\right)}={\text{MLP}}^{\left(l\right)}\left((1+\epsilon_{l})Z_{i}^{\left(l\right)}+\sum_{j\in N(i)}Z_{j}^{\left(l\right)}\right),\;i=1,2,\ldots ,N,$$

Where N(i) is the neighborhood of node *i*, $\epsilon_{l}$ is a learnable scalar, and ${\text{MLP}}^{\left(l\right)}(\cdot )$ is a multilayer perceptron equipped with ReLU activations and batch normalization.

#### 2.5.2 Projection and latent complex factors

The final GIN output is passed through a linear projection with ReLU and dropout to obtain a nonnegative factor matrix


(13) $$F\in R^{N\times M}.$$

The factor matrix *F* is used as a latent complex-membership representation learned by the graph autoencoder. We interpret $F_{i,m}$ as the membership strength of protein *i* to latent complex factor *m*.

#### 2.5.3 Complex extraction

To derive discrete complex predictions from the latent factor matrix F, each latent factor *m* was treated as a candidate complex seed, and proteins were ranked according to their membership scores $F_{i,m}$. Instead of using a single fixed complex size, we varied the top-*Q* selection size from Q=3 to Q=35 for each dataset. For each value of *Q*, the top-*Q* proteins with the highest scores in the *m*-th column of F were selected to form a candidate complex $C_{m,Q}$. Candidate complexes generated over all latent factors and all values of *Q* were then pooled to form the initial candidate set:


(14) $$C_{\text{raw}}=\overset{35}{\underset{Q=3}{\cup }}\overset{M}{\underset{m=1}{\cup }}C_{m,Q}.$$

This multi-*Q* extraction strategy was used to cover protein complexes with different subunit numbers, because protein complexes in curated references vary substantially in size. We did not tune a dataset-specific value of *Q*; instead, the same range Q=3,…,35 was used for all five benchmarks.

To obtain high-confidence predicted complexes, we further applied a size-adaptive filtering criterion based on the internal connectivity ratio. For a predicted protein set *P* with *m* proteins, let $L^{*}$ denote the number of observed PPI edges whose two endpoints both lie in *P*. The maximum possible number of internal edges is $\left(\begin{array}{c} m\\ 2 \end{array}\right)$, and the internal connectivity ratio is defined as


(15) $$R_{\text{conn}}(P)=\frac{L^{*}}{\left(\begin{array}{c} m\\ 2 \end{array}\right)}.$$

This metric quantifies the internal cohesiveness of a predicted complex in the PPI network, with larger values indicating stronger internal association.

Because the expected density of biologically plausible complexes depends on complex size, we used size-adaptive thresholds. For trimers and tetramers, only candidates with $R_{\text{conn}}\geq 1$ were retained, enforcing complete internal connectivity for small assemblies. For complexes with five or more subunits, we used a relaxed threshold of $R_{\text{conn}}\geq 0.3$, acknowledging that larger assemblies may have lower observed edge density due to incomplete PPI measurements while still representing plausible protein complexes. The retained candidates after this filtering step were used as the final predicted complexes for evaluation.

#### 2.5.4 Inner-product decoder and reconstruction

We reconstruct interactions by an inner-product decoder,


(16) $${\hat{A}}_{ij}=f_{i}f_{j}^{⊤},$$

Where $f_{i}$ is the *i*-th row of F and ${\hat{A}}_{ij}$ denotes the reconstructed affinity between proteins *i* and *j*.

#### 2.5.5 Unsupervised training objective

Let E denote the observed edge set and $\bar{E}$ denote the set of non-edges sampled from the complement of the adjacency matrix, that is, $E=\{(i,j)|A_{ij}=1\}$ and $\bar{E}=\{(i,j)|A_{ij}=0\}$. We optimize the following loss:


(17) $$L(F)=-\frac{1}{\left|E\right|}\sum_{(i,j)\in E}\;\text{log}\;\left(1-\text{exp}\;\left(-{\hat{A}}_{ij}\right)\right)+\frac{1}{\left|\bar{E}\right|}\sum_{(i,j)\in \bar{E}}{\hat{A}}_{ij}.$$

The first term encourages high reconstruction for observed interactions, whereas the second penalizes spurious similarity among non-edges, thereby preserving global sparsity.

### 2.6 GO-based functional coherence assessment

To quantify the functional consistency of the complexes predicted by PCIPG 2.0, we retrieved Gene Ontology (GO) annotations for all proteins in each complex and evaluated within-complex semantic relatedness across the three GO branches: Biological Process (BP), Molecular Function (MF) and Cellular Component (CC). The rationale is that proteins participating in similar biological programs or molecular activities tend to exhibit higher GO semantic similarity, and such concordance is frequently observed among bona fide complex subunits.

Pairwise semantic similarity between genes/proteins was computed with the GOSemSim framework (Yu 2020). For a complex containing *n* genes, we formed a similarity matrix $S\in R^{n\times n}$, where $S_{ab}$ denotes the semantic similarity between gene *a* and gene *b*. We summarized the overall GO coherence of the complex by a normalized matrix magnitude,


(18) $$\text{GOScore}=\frac{1}{n}{\left\|S\right\|}_{F}=\frac{1}{n}\sqrt{\sum_{a=1}^{n}\sum_{b=1}^{n}S_{ab}^{2}},$$

Where $||\cdot |{|}_{F}$ is the Frobenius norm.

As a negative-control baseline, we constructed random complex sets following previous studies (Qi *et al.* 2008, Wang *et al.* 2022). Briefly, proteins were sampled uniformly from the corresponding PPI network to form synthetic complexes while matching the empirical complex-size distribution of the curated gold-standard reference. To obtain stable estimates of the null distribution, we generated five random complexes per gold-standard complex (random: gold = 5:1) and computed GOScore in the same manner.

### 2.7 Evaluation metrics

We evaluated predicted complexes using Precision, Recall, F-score and accuracy (Acc). Let $P=\{p_{1},\ldots ,p_{\left|P\right|}\}$ denote the set of predicted complexes and $B=\{b_{1},\ldots ,b_{\left|B\right|}\}$ denote the gold-standard complexes. For a predicted complex *p* and a gold complex *b*, let $V_{p}$ and $V_{b}$ be their corresponding protein sets. We quantify their overlap using neighborhood affinity (NA),


(19) $$\text{NA}(p,b)=\frac{|V_{p}\cap V_{b}{|}^{2}}{\left|V_{p}|\cdot |V_{b}\right|}.$$

A predicted complex is considered to match a gold complex if NA(p,b)≥ϕ.

Let $N_{cp}$ be the number of predicted complexes that match at least one gold complex, and $N_{cb}$ be the number of gold complexes that are matched by at least one prediction. We define


(20) $$\text{Precision}=\frac{N_{cp}}{\left|P\right|},\;\text{Recall}=\frac{N_{cb}}{\left|B\right|},$$

And compute the *F*-score as the harmonic mean of precision and recall,


(21) $$F-\text{score}=\frac{2\cdot \text{Precision}\cdot \text{Recall}}{\text{Precision}+\text{Recall}}.$$

To summarize protein-level agreement, we report


(22) $$\text{Acc}=\sqrt{\text{Sn}\cdot \text{PPV}},$$

Where Sn and PPV are computed from the overlap matrix $T\in N^{\left|B|\times |P\right|}$ with entries


(23) $$T_{ij}=|V_{b_{i}}\cap V_{p_{j}}|,\;N_{i}=|V_{b_{i}}|.$$

Sensitivity (Sn) and positive predictive value (PPV) are defined as


(24) $$\text{Sn}=\frac{\sum_{i}{\text{max}}_{j}T_{ij}}{\sum_{i}N_{i}},\;\text{PPV}=\frac{\sum_{j}{\text{max}}_{i}T_{ij}}{\sum_{j}\sum_{i}T_{ij}}.$$

Gold-standard complexes were not used during multi-omics fusion, candidate-edge recovery, PPI network construction, structural feature extraction, graph autoencoder training, latent factor learning or complex extraction. After PCIPG 2.0 generated predicted complexes in an unsupervised manner, the curated reference complexes were used only to compute Precision, Recall, *F*-score and Acc.

Because different PPI benchmarks and structural-feature files cover different subsets of proteins, we applied an evaluation-coverage filtering step to the reference complexes. Let B denote the curated gold-standard complex set and let $V_{\text{model}}$ denote the set of proteins represented in the model input with available features. The evaluable reference set was defined as


(25) $$B_{\text{eval}}=\{B\in B|B\subseteq V_{\text{model}},\;|B|\geq 3\}.$$

Only $B_{\text{eval}}$ was used for final metric calculation. This step does not use gold-standard complexes to filter the input PPI network or train the model; it only excludes reference complexes that cannot be completely recovered because one or more of their subunits are absent from the model’s protein universe.

## 3 Results

### 3.1 PCIPG2.0 achieves leading performance on protein complex identification across datasets

We compared PCIPG with six state-of-the-art protein complex identification methods (see Supplementary Materials) on five widely used yeast benchmarks (Collins, Krogan-core, Krogan14k, DIP and BioGRID which describrd in Section 2.2) using Precision, Recall, *F*-score and Acc (see Section 2.7). These datasets span distinct network scales and experimental regimes, providing complementary tests under sparsity and noise.

**Figure 3:**
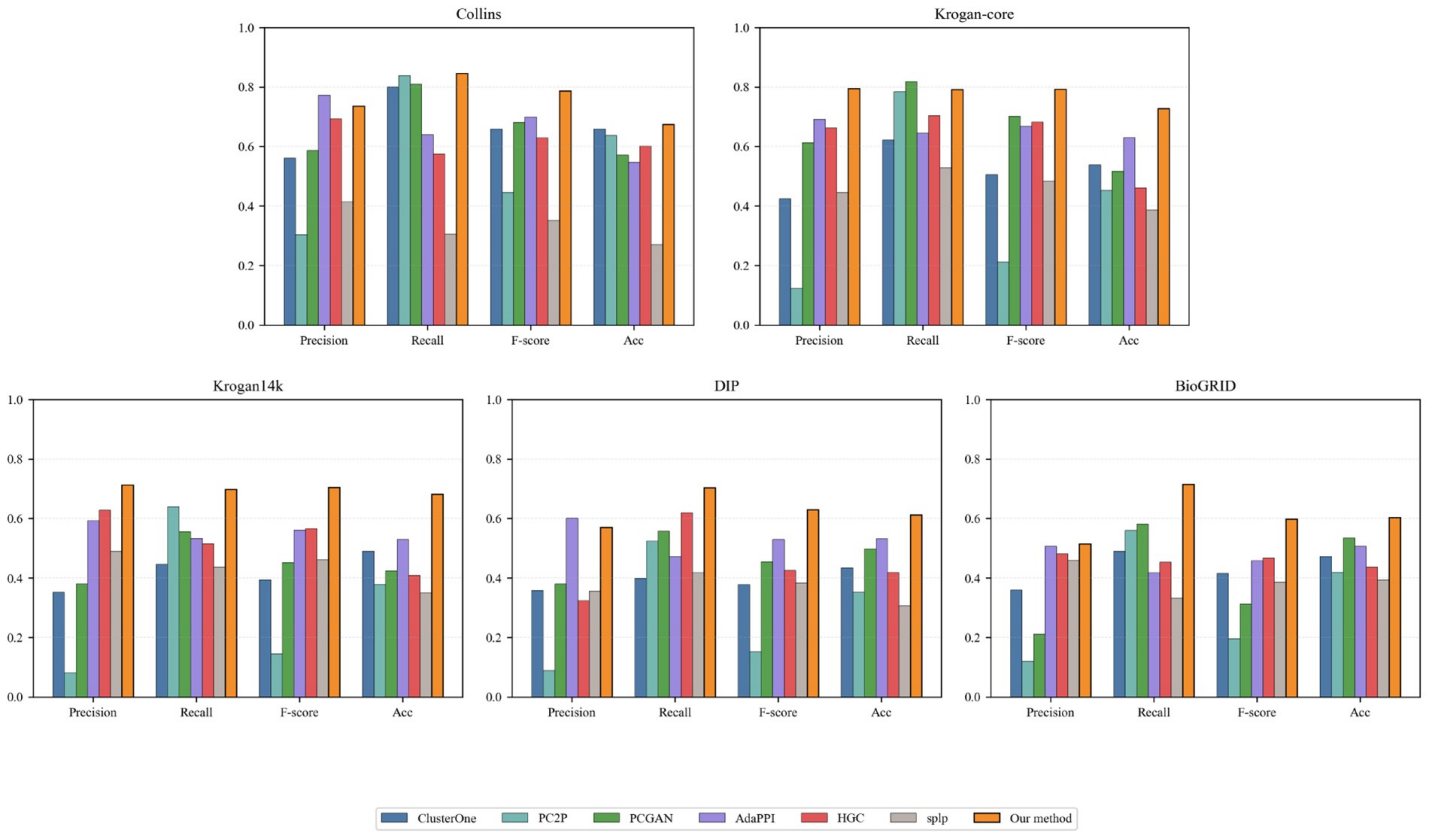
Performance comparison of protein complex identification methods across five yeast PPI benchmarks. Bar plots report Precision, Recall, *F*-score and Accuracy (Acc) on Collins, Krogan-core, Krogan14k, DIP and BioGRID for seven representative methods (ClusterONE, PC2P, PCGAN, AdaPPI, HGC, SPLP and our method; colors as indicated).

The results are shown in Fig. 3. Across all five benchmarks, PCIPG achieved the strongest overall performance, attaining the highest *F*-score and Acc relative to all compared baselines. Importantly, PCIPG maintained a balanced precision–recall profile across datasets, avoiding the common trade-off in which high-precision methods sacrifice coverage whereas recall-oriented methods introduce spurious complexes. Because the baselines span multiple methodological categories, including overlapping clustering, topology-driven representation learning and graph-based inference, these results suggest that the gains of PCIPG are not confined to a single comparison regime. To further assess whether the observed improvements were sensitive to stochastic training effects, we repeated PCIPG 2.0 20 times with different random seeds while keeping all hyperparameters fixed. The repeated-run analysis showed small standard deviations for *F*-score and Acc across all five benchmarks, indicating that the performance gains are stable and not attributable to a favorable random initialization. The full mean ± standard deviation results are provided in Table S4, available as supplementary data at *Bioinformatics* online.

These results further indicate that purely topology-driven clustering may generalize poorly across network regimes. By learning global latent representations through an encoder–decoder reconstruction objective, the graph autoencoder captures multi-hop interaction patterns beyond local graph density. Incorporating residue-derived structural features further strengthens node representations by encoding intra-protein constraints that are not accessible from topology alone. The strongest improvements were observed in F-score and Acc rather than in recall alone, suggesting that the method improves prediction purity while preserving coverage, rather than merely increasing the number of predicted clusters.

### 3.2 Impact of multi-omics–driven network enhancement

**Figure 4:**
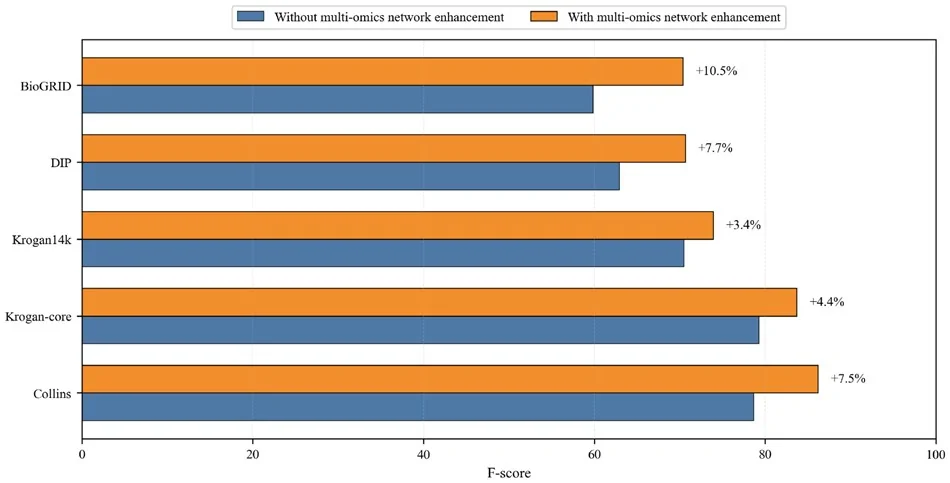
Multi-omics–driven network enhancement improves protein complex identification across yeast PPI benchmarks. Horizontal bars compare *F*-score for PCIPG without network enhancement (PCIPG2.0-Base) versus with multi-omics enhancement (PCIPG2.0-Full) on Collins, Krogan-core, Krogan14k, DIP and BioGRID. Multi-omics enhancement yields consistent gains across all datasets, with the largest improvements on noisier, larger-scale networks (DIP and BioGRID).

We quantified the contribution of the multi-omics fusion strategy by comparing the base model without network enhancement (PCIPG2.0-Base) with the full model incorporating multi-omics enhancement (PCIPG2.0-Full) across all five benchmarks in Fig. 4.

Multi-omics enhancement consistently improved performance on every dataset, with the most pronounced gains observed on larger and noisier interaction maps such as DIP and BioGRID. These results support the premise that complementary omics evidence can recover plausible functional associations that are obscured by missing or noisy PPIs, thereby facilitating downstream complex discovery.

Analysis of Precision and Recall showed that the improvement was driven primarily by increased Precision while maintaining comparable Recall (Table S1, available as supplementary data at *Bioinformatics* online). This pattern suggests that network enhancement improves the effective completeness of the graph by prioritizing likely missing associations, rather than simply increasing the number of recovered clusters at the cost of additional false positives. In other words, the added candidate edges appear to improve graph quality in a way that benefits complex purity while preserving coverage.

To further assess the robustness of this strategy, we examined the sensitivity of performance to the number of added candidate edges *P*. Over a moderate range of *P* values, PCIPG 2.0 showed stable performance, with the default setting P=200 providing a favorable balance between graph completion and false-edge introduction (Table S2, available as supplementary data at *Bioinformatics* online). When *P* was too small, the enhancement effect became limited; when *P* was too large, excessive candidate edges slightly reduced Precision on the sparsest benchmarks. These observations are consistent with the interpretation that multi-omics enhancement is most effective when it selectively augments plausible missing interactions without overwhelming the original graph structure.

We next compared PCIPG 1.0 and PCIPG 2.0 directly (Table 2). PCIPG 2.0 improved overall performance across all five benchmarks, yielding consistently higher F-scores and increased Acc on every dataset. In most cases, the gains were driven by higher Precision with largely preserved Recall, indicating that the updated model produces cleaner complex assignments without compromising coverage. Taken together, these results indicate that multi-omics–driven interactome completion is not merely an auxiliary preprocessing step, but a major source of performance improvement in PCIPG 2.0.

**Table 2:** Performance comparison between PCIPG 1.0 and PCIPG 2.0 across five yeast PPI benchmarks.

Dataset	Precision	Recall	*F*-score	Acc
Collins	0.7799/**0.8292**	0.8939/**0.8971**	0.8330/**0.8618**	0.6461/**0.6795**
Krogan-core	0.8407/**0.8597**	0.7969/**0.8154**	0.8182/**0.8369**	0.6585/**0.7370**
Krogan14k	0.7359/**0.7632**	0.7139/**0.7165**	0.7247/**0.7391**	0.6155/**0.6923**
DIP	0.6489/**0.6753**	**0.7440**/0.7406	0.6932/**0.7064**	0.5776/**0.6173**
BioGRID	0.5948/**0.6215**	**0.8155**/0.8116	0.6879/**0.7039**	0.5880/**0.6280**

In each cell, the value before the slash is PCIPG 1.0 and the value after the slash is PCIPG 2.0.

Bold values indicate the better-performing results for each metric.

### 3.3 Ablation study and robustness to architectural choices

To better isolate the contribution of each module, we performed two types of ablation analyses: component-level ablations and architecture-replacement ablations.

First, removing the multi-omics enhancement module led to a systematic decrease in performance across all five benchmarks, confirming that candidate-edge recovery is an important source of improvement under incomplete network measurements. Second, removing the structure-aware residue-derived node features also reduced performance substantially. In several datasets, the decrease was especially pronounced in Acc, indicating that residue-level structural cues improve not only overall complex recovery but also the protein-level agreement between predicted and reference complexes. Third, when both multi-omics enhancement and structure-aware node features were removed, performance decreased further across all benchmarks, supporting that these two modules make complementary rather than redundant contributions to complex identification (Table S2, available as supplementary data at *Bioinformatics* online).

To assess robustness with respect to architectural choices, we further performed controlled module-replacement analyses (Table 3). Replacing the ProteinMPNN encoder with alternative residue feature extractors led to a consistent drop in performance, indicating that the learned structural embeddings contribute substantially beyond generic structural descriptors. Likewise, substituting the PPI encoder from GIN to other message-passing backbones reduced *F*-score and Acc, suggesting that the GIN-based aggregation is particularly effective at capturing multi-hop interaction patterns needed for complex discovery. Finally, when the protein representation module’s GCN, which aggregates residue-level embeddings into protein-level node features, was replaced by non-graph alternatives, performance deteriorated further, underscoring the importance of explicitly modeling intra-protein structural dependencies before PPI-level learning.

**Table 3:** Module substitution study.

Abl.	Variant	Collins	Krogan-core	Krogan14k	DIP	BioGRID
RFE	ESM	0.8240/0.6520	0.8010/0.7030	0.7080/0.6580	0.6620/0.5790	0.6540/0.5890
RFE	PhysOH	0.7920/0.6240	0.7680/0.6750	0.6760/0.6320	0.6180/0.5440	0.6100/0.5560
RFE	Rand	0.7420/0.5850	0.7160/0.6350	0.6280/0.5880	0.5620/0.4980	0.5480/0.5100
PPIE	GCN	0.8180/0.6480	0.7920/0.7010	0.7020/0.6560	0.6580/0.5780	0.6490/0.5890
PPIE	SAGE	0.8260/0.6550	0.8010/0.7100	0.7100/0.6620	0.6660/0.5850	0.6570/0.5960
PPIE	GAT	0.8040/0.6360	0.7780/0.6880	0.6900/0.6450	0.6420/0.5660	0.6330/0.5770
PRA	MeanPool	0.8000/0.6280	0.7760/0.6820	0.6860/0.6380	0.6200/0.5440	0.6060/0.5560
PRA	SeqTrans	0.8120/0.6380	0.7880/0.6940	0.6960/0.6480	0.6340/0.5580	0.6200/0.5690
PRA	MLPpool	0.7760/0.6080	0.7520/0.6620	0.6640/0.6170	0.5980/0.5220	0.5820/0.5360
–	**PCIPG 2.0**	**0.8618**/**0.6795**	**0.8369**/**0.7370**	**0.7391**/**0.6923**	**0.7064**/**0.6173**	**0.7039**/**0.6280**

We report *F*-score/Acc on five yeast PPI benchmarks. Ablations include: *RFE*, residue feature extractor; *ESM*, PhysOH, Rand; *PPIE*, PPI encoder: GCN, SAGE, GAT; *PRA*, protein representation aggregator: MeanPool, SeqTrans, MLPpool.

Bold values indicate the better-performing results for each dataset.

Together, these ablations show that PCIPG 2.0 benefits from two distinct but complementary mechanisms: improved graph completeness through multi-omics–driven candidate-edge recovery, and improved biological specificity through residue-informed protein representations. The overall performance gain therefore arises from the interaction of graph completion, structure-aware representation learning and global PPI-context modeling, rather than from any single component in isolation.

Although experimentally resolved structures provide the most reliable structural templates, they are available for only a subset of proteins. Therefore, structure-aware analyses often need to rely on predicted structural models. To examine whether PCIPG 2.0 depends critically on experimentally solved structures, we performed a structure-source robustness analysis in which all experimentally resolved structures were replaced by AlphaFold-predicted models across the five *Saccharomyces cerevisiae* PPI benchmarks.

As shown in Table 4, replacing experimentally resolved structures with predicted models led to a moderate but not catastrophic decrease in performance across datasets. Averaged over the five benchmarks, the predicted-only setting decreased Precision from 0.7498 to 0.7010, Recall from 0.7962 to 0.7714, *F*-score from 0.7696 to 0.7316 and Acc from 0.6708 to 0.6310. The larger reductions in Precision and Acc suggest that experimentally resolved structures provide cleaner residue-level contact information and help improve the purity and protein-level agreement of predicted complexes. In contrast, Recall was less affected, indicating that the use of predicted structures did not substantially reduce the ability of PCIPG 2.0 to recover gold-standard complexes.

**Table 4:** Robustness of PCIPG 2.0 to structural source across five *Saccharomyces cerevisiae* PPI benchmarks.

Dataset	Structure source	Precision	Recall	*F*-score	Acc
**BioGRID**	Predicted-only	0.5750	0.7900	0.6656	0.5900
	Default	0.6215	0.8116	0.7039	0.6280
**Collins**	Predicted-only	0.7850	0.8720	0.8262	0.6450
	Default	0.8292	0.8971	0.8618	0.6795
**DIP**	Predicted-only	0.6250	0.7200	0.6691	0.5750
	Default	0.6753	0.7406	0.7064	0.6173
**Krogan-core**	Predicted-only	0.8150	0.7850	0.7997	0.6950
	Default	0.8597	0.8154	0.8369	0.7370
**Krogan14k**	Predicted-only	0.7050	0.6900	0.6974	0.6500
	Default	0.7632	0.7165	0.7391	0.6923

“Default” denotes the standard PCIPG 2.0 setting in which experimentally resolved structures are used when available and predicted structures are used otherwise. “Predicted-only” denotes the control setting in which all experimentally resolved structures are replaced by AlphaFold-predicted models.

These results suggest that PCIPG 2.0 benefits from experimentally resolved structures but does not depend critically on them. Predicted structures still provide useful residue-level constraints for complex inference, allowing the framework to retain competitive performance even when experimentally solved structures are unavailable. At the same time, the moderate decline observed in the predicted-only setting indicates that structural uncertainty should be considered when interpreting individual predicted complexes, especially those dominated by low-confidence predicted models.

### 3.4 GO coherence analysis supports the functional plausibility of PCIPG 2.0-predicted complexes

To assess whether PCIPG 2.0-predicted complexes exhibit biologically coherent functional signals beyond network topology, we quantified Gene Ontology (GO) semantic similarity among proteins within each predicted complex across five yeast PPI benchmarks (BioGRID, Collins, DIP, Krogan14k and Krogan-core). For each dataset, we computed within-complex semantic similarity scores separately for GO Biological Process (BP), Cellular Component (CC) and Molecular Function (MF), and compared three complex types: curated gold-standard complexes (*Known*), PCIPG 2.0 predictions (*Predicted*) and size-matched random protein sets (*Random*).

**Figure 5:**
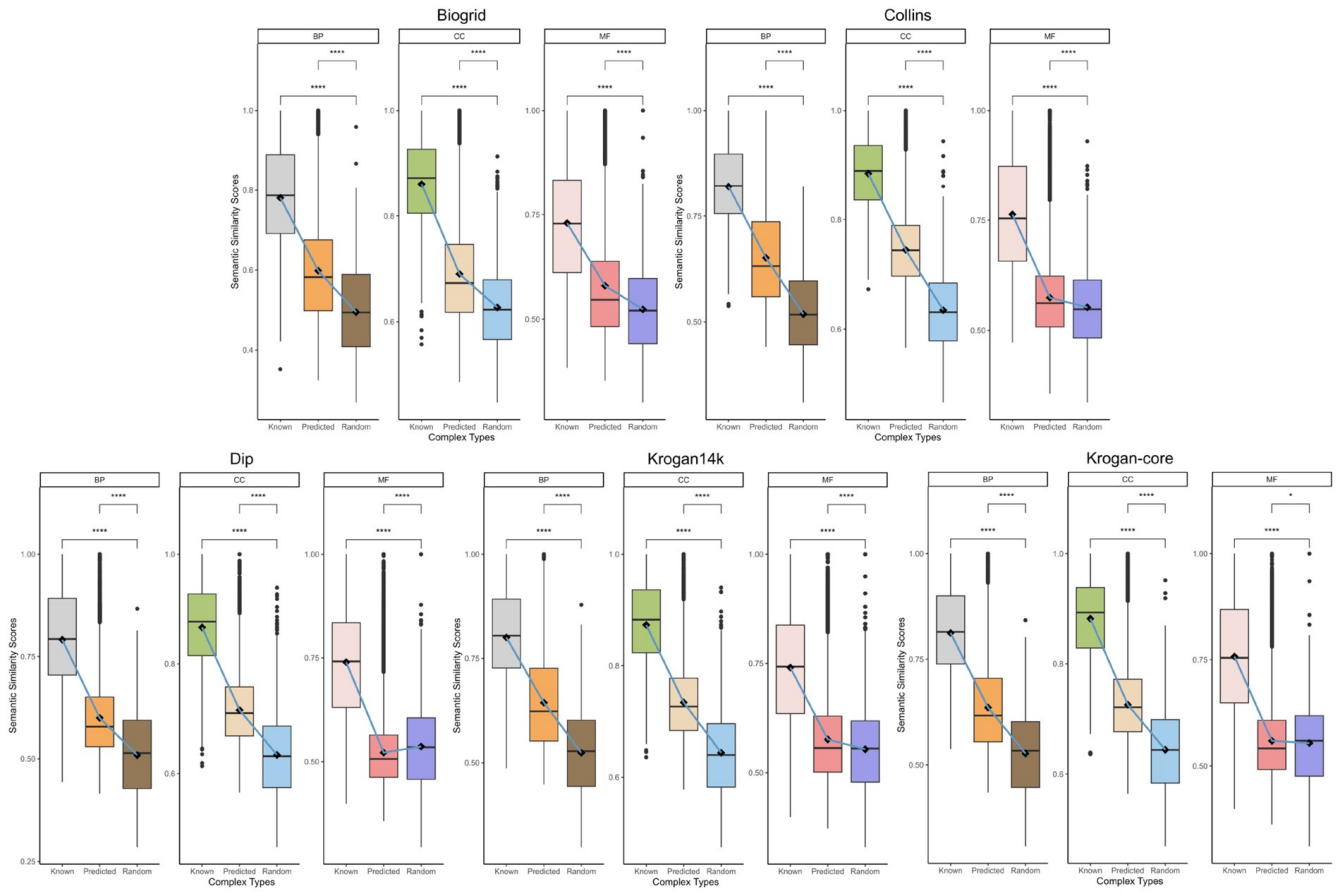
GO semantic similarity analysis of PCIPG 2.0-predicted complexes across five yeast PPI benchmarks. Within-complex GO semantic similarity scores are shown for curated gold-standard complexes (*Known*), PCIPG 2.0-predicted complexes (*Predicted*) and size-matched random protein sets (*Random*) on BioGRID, Collins, DIP, Krogan14k and Krogan-core. For each dataset, similarity distributions are reported for GO Biological Process (BP), Cellular Component (CC) and Molecular Function (MF). Box plots indicate the median and interquartile range; whiskers denote 1.5×IQR and points denote outliers. Blue lines connect group means. Brackets indicate pairwise statistical comparisons between complex types (asterisks denote significance; ${\;}^{*}P<0.05$, ${\;}^{**}P<0.01$, ${\;}^{***}P<0.001$, ${\;}^{****}P<0.0001$).

Across all five benchmarks and all three ontologies, PCIPG 2.0 predictions consistently showed significantly higher GO similarity than random sets, indicating non-random functional coherence of the recovered assemblies (Fig. 5). As expected, gold-standard complexes typically achieved the highest similarity, whereas predicted complexes exhibited intermediate coherence, reflecting substantial shared biological roles together with dataset-specific noise, incomplete annotation and partial recovery of subunits. Notably, the separation between *Predicted* and *Random* was most pronounced for GO Biological Process, indicating that PCIPG 2.0 preferentially recovers complexes whose members participate in shared biological programs. In contrast, GO Cellular Component showed the smallest gap between *Predicted* and *Random*, suggesting that subcellular co-localization signals are comparatively weaker and may be more susceptible to incomplete localization annotations or context-dependent compartmentalization.

This overall pattern remained consistent across interactomes of markedly different scale and density. Together, these GO-based results provide systematic support for the functional plausibility of the predicted complexes. Because GO similarity primarily captures functional relatedness rather than direct physical assembly, we next complemented this systems-level analysis with literature-supported case studies and AlphaFold3-based structural modeling.

### 3.5 PCIPG 2.0 recovers biologically coherent complexes involved in key cellular processes

As a representative literature-supported case, PCIPG 2.0 identified a *CDC23*-centred assembly consisting of *YLR127C*, *YLR102C*, *YGL240W*, *YIR025W*, *YOR249C*, *YKL022C*, *YNL172W*, *YOL069W*, *YPL174C* and *YHR166C* (*CDC23*). This prediction overlaps substantially with a literature-curated anaphase-promoting complex/cyclosome (APC/C) subcomplex, sharing 8 of 13 reported components. Given the central role of APC/C in cell-cycle control, this result suggests that PCIPG 2.0 can recover functionally coherent modules even when some annotated subunits are not fully captured (Bodrug *et al.* 2023, Darling *et al.* 2024, Höfler *et al.* 2024, Sitry-Shevah *et al.* 2024, Vazquez-Fernandez *et al.* 2024, Paul *et al.* 2025, Skrajna *et al.* 2025).

PCIPG 2.0 also recovered a highly coherent octameric assembly composed of *YPR108W* (Rpn7), *YOR261C* (Rpn8), *YLR421C* (Rpn13), *YOR259C* (Rpt4), *YOR117W* (Rpt5), *YKL145W* (Rpt1), *YMR314W* (Pre5) and *YOL038W* (Pre6), consistent with a proteasome-associated module spanning both the 19S regulatory particle and the 20S core particle of the 26S proteasome. The fact that all predicted members map to a well-characterized conserved machinery supports that the method can recover biologically central assemblies with high internal consistency (Zhang *et al.* 2022, Arkinson *et al.* 2025, Draczkowski *et al.* 2025, Gao *et al.* 2025).

Together, these representative examples indicate that the complexes recovered by PCIPG 2.0 are not merely topological co-clusters, but biologically coherent modules supported by prior mechanistic knowledge.

**Figure 6:**
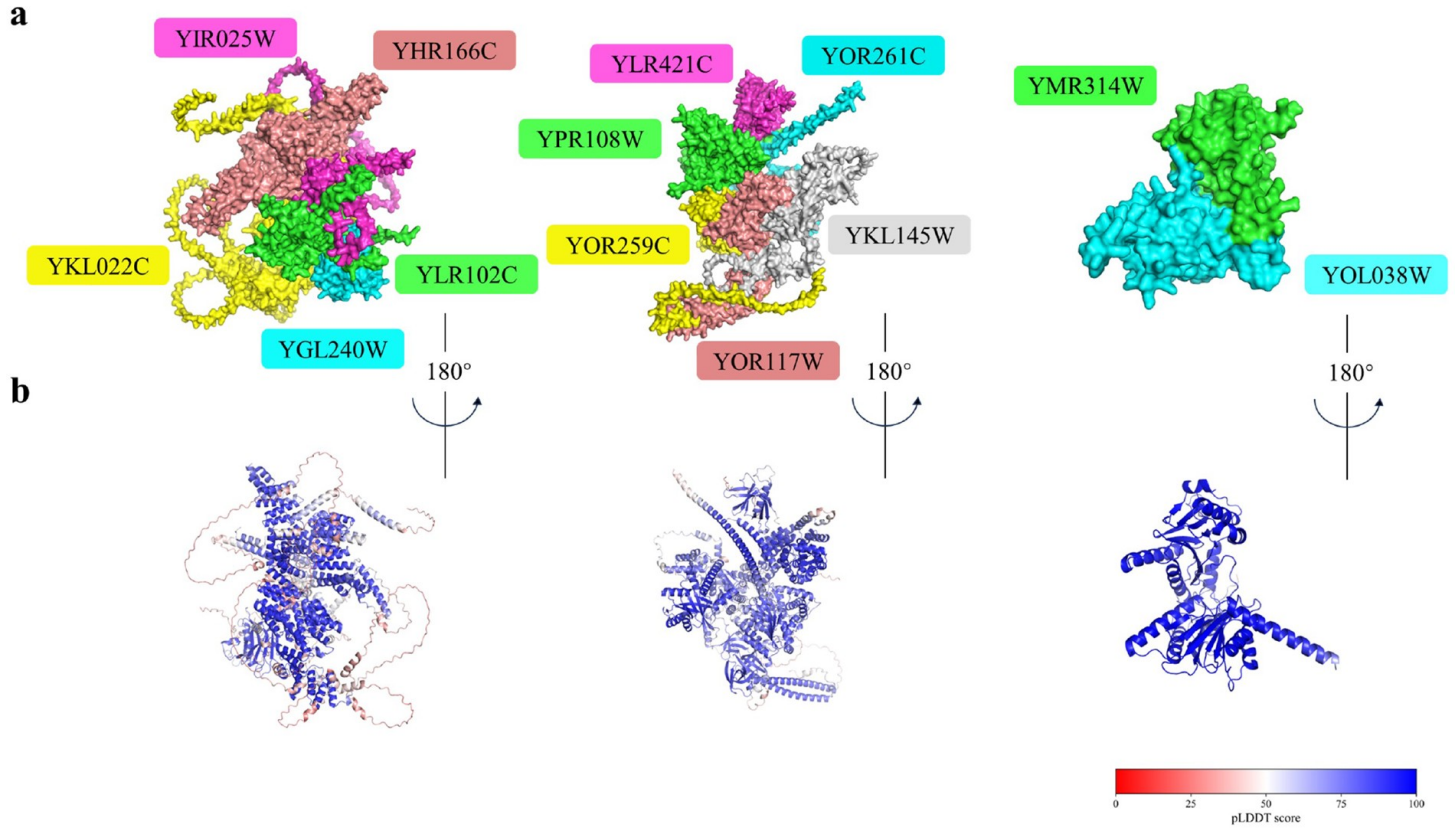
AlphaFold3 assembly of representative PCIPG 2.0-predicted complexes. (a) AF3-predicted sub-assemblies rendered in surface representation and coloured by chain. Left, APC/C TPR-module subcomplex: *YKL022C* (Cdc16/Apc6), *YHR166C* (Cdc23/Apc8), *YLR102C* (Apc9), *YIR025W* (Mnd2) and *YGL240W* (Doc1/Apc10). Middle, 26S proteasome 19S-associated module: *YOR259C* (Rpt4), *YOR117W* (Rpt5), *YKL145W* (Rpt1), *YPR108W* (Rpn7), *YOR261C* (Rpn8) and *YLR421C* (Rpn13). Right, 20S core module: *YMR314W* (Pre5) and *YOL038W* (Pre6). (b) The same models coloured by per-residue confidence (pLDDT; scale shown). For visualization, each complex is shown in two orientations related by a $180^{{}^{\circ}}$ rotation.

To further substantiate the structural plausibility of PCIPG 2.0-predicted complexes, we performed multimeric structure prediction using AlphaFold3 (Abramson *et al.* 2024) for these representative assemblies: the 26S proteasome-associated complex and the APC/C module. Because AF3 imposes practical limits on the total sequence length of multichain inputs, we partitioned the 26S proteasome prediction into two structurally coherent sub-assemblies and predicted them separately: (i) a 19S regulatory particle module comprising *YOR259C* (Rpt4), *YOR117W* (Rpt5), *YKL145W* (Rpt1), *YPR108W* (Rpn7), *YOR261C* (Rpn8) and *YLR421C* (Rpn13); and (ii) a 20S core particle module comprising *YMR314W* (Pre5) and *YOL038W* (Pre6). Both yielded tightly packed assemblies with well-formed inter-chain interfaces in surface renderings (Fig. 6a), and the corresponding per-residue confidence profiles (pLDDT) indicated predominantly high-confidence cores with lower-confidence regions largely confined to flexible segments (Fig. 6b). These results support that the PCIPG 2.0 predictions are not merely topological co-clusters, but exhibit a strong tendency to form physically compatible assemblies consistent with the known modular organization of the 26S proteasome.

Similarly, for the APC/C case, AF3 modelling of a length-feasible subcomplex, *YKL022C* (Cdc16/Apc6), *YHR166C* (Cdc23/Apc8), *YLR102C* (Apc9), *YIR025W* (Mnd2) and *YGL240W* (Doc1/Apc10), produced a compact structure centred on the TPR scaffold (Cdc16–Cdc23) with additional regulatory/adaptor subunits arranged in close spatial proximity (Fig. 6). The ability of AF3 to assemble these modules with coherent interfaces provides orthogonal structural support for the biological credibility of the complexes recovered by PCIPG 2.0 and further reinforces their functional interpretability.

## 4 Discussion

PCIPG 2.0 is a fully unsupervised framework that combines multi-omics–driven interactome completion with structure-aware node representations for protein complex discovery. To make the relationship between PCIPG 1.0 and PCIPG 2.0 explicit, we distinguish the advance of PCIPG 2.0 at three levels. Conceptually, PCIPG 2.0 moves beyond topology-centered complex detection by treating protein complex identification as a multi-scale inference problem under incomplete interactome measurements, where missing PPI edges and residue-level structural determinants are addressed jointly. Architecturally, this conceptual shift is realized by two major additions to the original PCIPG framework: a multi-omics fusion module for candidate-edge recovery and a residue-informed structural representation module for generating protein-level node features, both of which are integrated into the PPI-level graph autoencoder for unsupervised complex prediction. Empirically, our ablation results indicate that multi-omics-based interactome completion is the main source of the observed performance gain in practice, because it consistently improves *F*-score across all five benchmarks and mainly increases Precision while maintaining comparable Recall. The structure-aware representation module provides a complementary contribution by improving protein-level agreement and biological specificity, especially when combined with the enhanced PPI graph. Thus, PCIPG 2.0 should be viewed not simply as a larger implementation of PCIPG 1.0, but as an extension that couples graph completion, residue-informed representation learning and global PPI-context modeling within a unified unsupervised framework. By integrating cross-view graph fusion in Stage I and residue-informed PPI representation learning in Stage II, it improves complex recovery over PCIPG 1.0 across five yeast benchmarks, with consistent gains in *F*-score and Acc.

Several limitations remain. The interactome completion stage relies on informative and well-aligned multi-omics data, and its reliability may decrease when omics views are missing or mismatched to the PPI source. Although the structure-aware module improves complex identification, its reliability depends on the quality of the input structural models. Experimentally resolved structures are expected to provide more reliable residue-level contact graphs, whereas predicted structures may contain local errors, uncertain flexible regions or inaccurate domain orientations. Our stratified analysis suggests that complexes involving predicted structures are not systematically low-confidence, but structural uncertainty should still be considered when interpreting individual predictions. In future work, confidence-aware structural encoding, for example by weighting residue contacts or protein-level structural features according to pLDDT, PAE or related uncertainty estimates, may further improve the robustness of PCIPG 2.0. In addition, the current factor-based complex extraction strategy may under-recover very large or transient assemblies, and GO-based validation shows branch-dependent signal strength, with weaker effects in Cellular Component. We also note that the GO semantic similarity and AlphaFold3-based assembly analyses should be interpreted as supportive computational evidence rather than definitive validation of physical complex formation. These analyses help assess whether predicted complexes are functionally coherent and structurally plausible, but biochemical or proteomic experiments will be required to confirm newly proposed assemblies.

This study is limited to *Saccharomyces cerevisiae* and focuses on static complex discovery, leaving cross-species generalization and condition-specific assembly dynamics unresolved. Future work will focus on uncertainty-aware fusion, handling missing or condition-specific omics views, and incorporating multi-chain structural modeling to better capture higher-order assembly constraints.

## 5 Conclusion

PCIPG 2.0 establishes an unsupervised framework for protein complex identification by explicitly combining multi-omics–driven interactome completion with structure-aware protein representation learning. By integrating residue-derived structural cues and multi-omics functional signals, the framework helps alleviate two common limitations of PPI-based inference: missed interactions and limited mechanistic resolution. Through multi-view Laplacian-consistent fusion and a structure-aware PPI-level graph autoencoder, PCIPG 2.0 delivers robust gains across yeast benchmarks and produces complexes with non-random functional coherence. Together, these results suggest that PCIPG 2.0 provides a conceptually grounded and extensible route to recovering biologically meaningful assemblies from incomplete and noisy interactomes, while offering a modular foundation for incorporating richer omics evidence and structural signals in future complex-discovery studies.

## Supplementary Material

btag552_Supplementary_Data

## Data Availability

The source code is available at GitHub: https://github.com/hyx-1/PCIPG2.0. The complete reproducibility package, including the code, processed data, configuration files and materials required to reproduce the experiments reported in this manuscript, has been archived on Zenodo with an archival DOI: https://doi.org/10.5281/zenodo.20133228. The GitHub repository also provides the README-based usage instructions.
