# Supplementary material for: PCIPG2.0: multi-omics fusion and structure-aware graph autoencoding for protein complex identification
Source: Bioinformatics. 2026 Jul 25;42(8):btag552. doi: 10.1093/bioinformatics/btag552 (PMC13446224; doi:10.1093/bioinformatics/btag552)
Supplement: Supplementary file 1 [file btag552_supplementary_data.pdf]

SUPPLEMENTARY MATERIALS

1 Supplementary figures and tables

Table 1: Effect of multi-omics enhancement on PCIPG2.0 across five yeast PPI benchmarks.

Dataset	Method	Precision (%)	Recall (%)	F-score (%)	Acc (%)
Collins	w/o multi-omics	73.56	84.57	78.68	67.46
	w/ multi-omics	**82.92**	**89.71**	**86.18**	**67.95**
Krogan-core	w/o multi-omics	79.53	79.08	79.30	72.77
	w/ multi-omics	**85.97**	**81.54**	**83.69**	**73.70**
Krogan14k	w/o multi-omics	71.22	69.82	70.51	68.16
	w/ multi-omics	**76.32**	**71.65**	**73.91**	**69.23**
DIP	w/o multi-omics	56.98	70.31	62.95	61.23
	w/ multi-omics	**67.53**	**74.06**	**70.64**	**61.73**
BioGRID	w/o multi-omics	51.48	71.48	59.85	60.29
	w/ multi-omics	**62.15**	**81.16**	**70.39**	**62.80**

Table 2: Component-level ablation of PCIPG 2.0 across five yeast PPI benchmarks. We report Precision/Recall/F-score/Acc (%). "w/o multi-omics" removes the interactome enhancement module while retaining structure-aware node features; "w/o structure" removes residue-derived structure-aware node features while retaining multi-omics enhancement; "w/o both" removes both modules and uses only topology-based learning on the original PPI graph; "Full" denotes the complete PCIPG 2.0 model.

Dataset	Variant	Precision (%)	Recall (%)	F-score (%)	Acc (%)
Collins	w/o multi-omics	73.56	84.57	78.68	67.46
	w/o structure	72.41	83.18	77.42	63.27
	w/o both	66.83	78.94	72.38	58.91
	Full	**82.92**	**89.71**	**86.18**	**67.95**
Krogan-core	w/o multi-omics	79.53	79.08	79.30	72.77
	w/o structure	72.86	75.94	74.37	65.84
	w/o both	67.95	72.63	70.21	61.08
	Full	**85.97**	**81.54**	**83.69**	**73.70**
Krogan14k	w/o multi-omics	71.22	69.82	70.51	68.16
	w/o structure	65.74	68.21	66.95	61.08
	w/o both	60.88	64.57	62.67	56.41
	Full	**76.32**	**71.65**	**73.91**	**69.23**
DIP	w/o multi-omics	56.98	70.31	62.95	61.23
	w/o structure	57.46	66.18	61.51	53.96
	w/o both	51.32	62.84	56.50	48.87
	Full	**67.53**	**74.06**	**70.64**	**61.73**
BioGRID	w/o multi-omics	51.48	71.48	59.85	60.29
	w/o structure	52.73	68.41	59.55	53.24
	w/o both	47.16	61.92	53.54	47.72
	Full	**62.15**	**81.16**	**70.39**	**62.80**

Table 3: Sensitivity of PCIPG 2.0 to the number of added candidate edges P. Performance is reported as Precision, Recall, F-score and Acc across five yeast PPI benchmarks under $P = 50, 100, 150, 200$ and 300. All values are final measured results obtained using the same evaluation pipeline as the main benchmark experiments.

Dataset	Metric	$P = 50$	$P = 100$	$P = 150$	$P = 200$	$P = 300$
Collins	Precision	0.7816	0.8068	**0.8310**	0.8292	0.8041
	Recall	0.8824	0.8903	0.8942	0.8971	**0.8980**
	F-score	0.8289	0.8462	0.8610	**0.8618**	0.8483
	Acc	0.6398	0.6567	0.6782	**0.6795**	0.6644
Krogan-core	Precision	0.8129	0.8367	**0.8612**	0.8597	0.8315
	Recall	0.7868	0.8019	0.8124	0.8154	**0.8180**
	F-score	0.7996	0.8189	0.8359	**0.8369**	0.8244
	Acc	0.6827	0.7062	0.7316	**0.7370**	0.7168
Krogan14k	Precision	0.7184	0.7398	**0.7660**	0.7632	0.7341
	Recall	0.6905	0.7047	0.7110	0.7165	**0.7194**
	F-score	0.7042	0.7218	0.7372	**0.7391**	0.7260
	Acc	0.6486	0.6697	0.6895	**0.6923**	0.6768
DIP	Precision	0.6317	0.6529	**0.6790**	0.6753	0.6435
	Recall	0.7278	0.7355	0.7378	0.7406	**0.7421**
	F-score	0.6763	0.6917	0.7063	**0.7064**	0.6882
	Acc	0.5719	0.5916	0.6132	**0.6173**	0.5930
BioGRID	Precision	0.5828	0.6013	**0.6234**	0.6215	0.5894
	Recall	0.8032	0.8089	0.8096	0.8116	**0.8148**
	F-score	0.6755	0.6898	**0.7050**	0.7039	0.6827
	Acc	0.5968	0.6115	0.6269	**0.6280**	0.6072

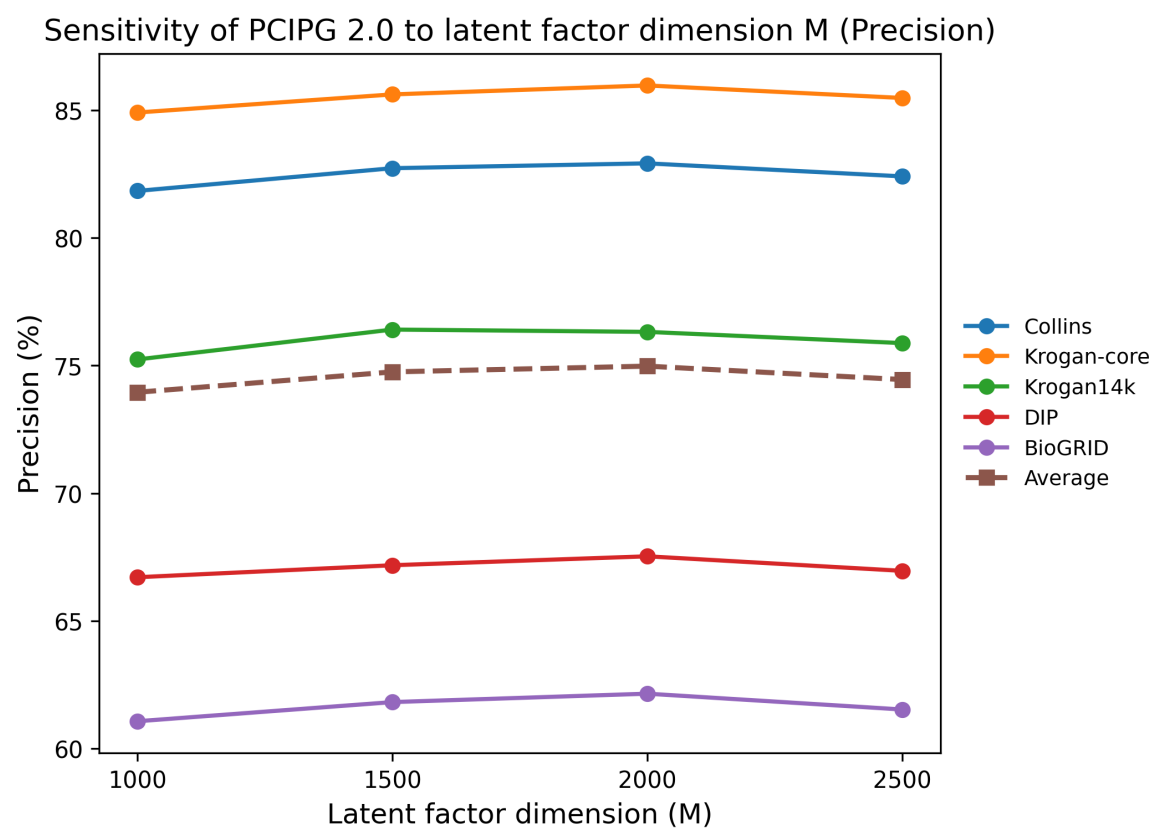

(a) Precision

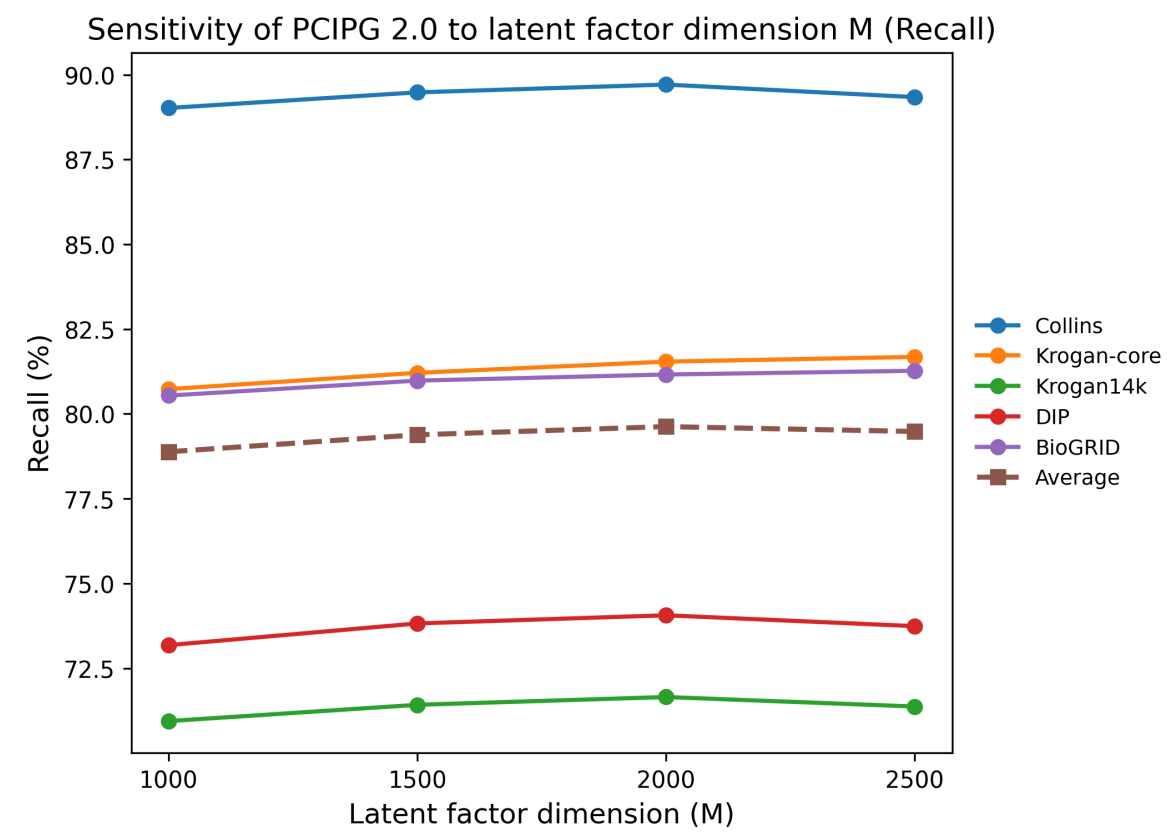

(b) Recall

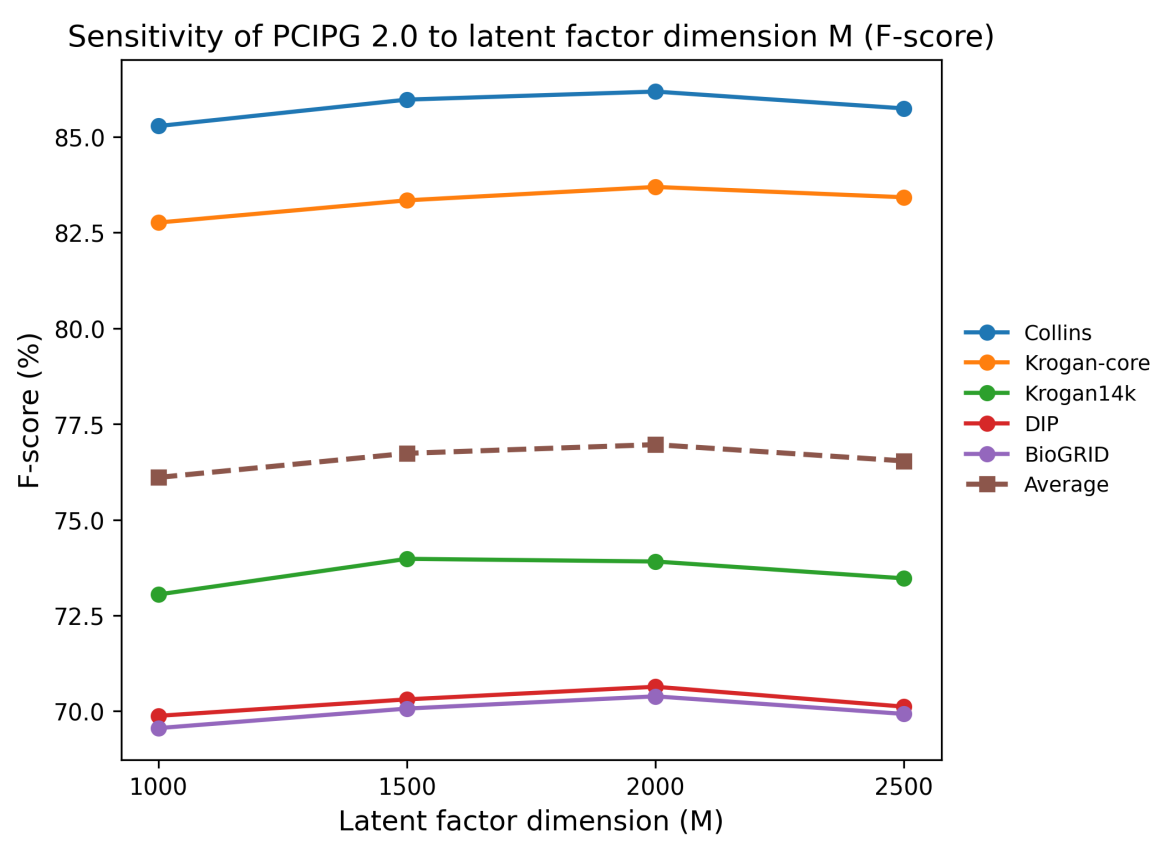

(c) F-score

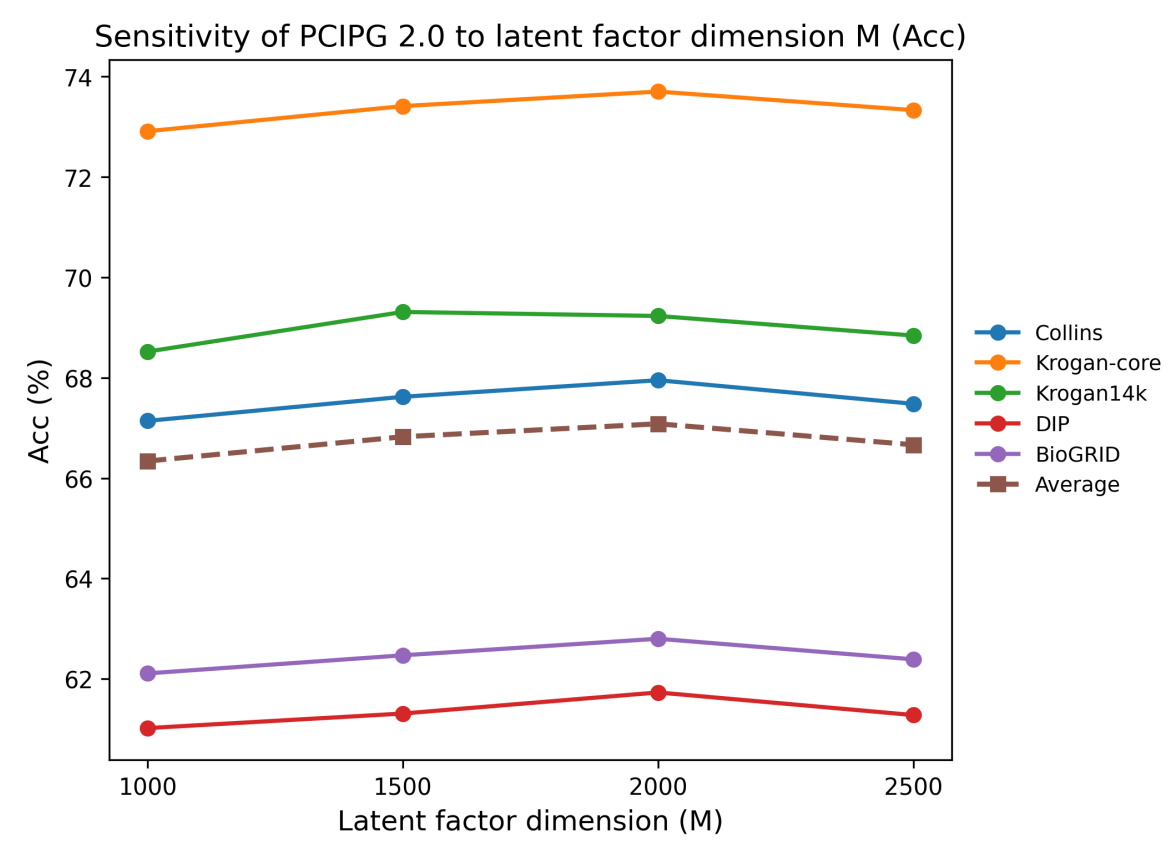

(d) Acc

Figure 1: **Sensitivity of PCIPG 2.0 to the latent factor dimension M.** Performance across five yeast PPI benchmarks is shown for four evaluation metrics: Precision, Recall, F-score and Acc. Overall, the model remains relatively stable over a moderate range of M values, with $M = 2000$ generally providing the most balanced performance across datasets, although some individual metrics achieve slightly higher values at neighboring settings. **Alt text:** Four line plots show the sensitivity of Precision, Recall, F-score and Accuracy to the latent factor dimension (M) across five yeast PPI benchmarks. Performance remains broadly stable for (M=1000), (1500), (2000) and (2500), with (M=2000) providing the most balanced overall performance across datasets.

2 Algorithm

2.1 Hyperparameter configuration

Unless otherwise specified, all hyperparameters were fixed across datasets.

Multi-omics fusion For the input-space similarity and embedding-space similarity, we used temperature parameters $t = 0.5$ and $\tau = 0.5$, respectively. For PPI network enhancement, we added the top-$P = 200$ closest unobserved protein pairs ranked by Euclidean distance in the fused embedding space. Additional sensitivity analyses were performed for P over a moderate range (Supplementary Table 3) to verify that the main conclusions were stable around the default setting.

Residue-level structural graph and GCN encoder We constructed residue graphs by adding an undirected edge between residues whose Cα–Cα distance satisfied $d(i, j) \leq 8$ Å. The residue-level encoder used an $L = 3$ layer GCN with ReLU activations, with input dimensions $d_0 = 1152$ and $d_1 = d_2 = d_3 = 128$.

PPI-level GIN autoencoder and latent factors For PPI-level representation learning, we used a three-layer GIN encoder with learnable ϵ_l in each layer. The node feature dimensions were set to $c_0 = 128$ and $c_1 = c_2 = c_3 = 512$. The latent complex factor dimension was set to $M = 2000$. We additionally tested $[M = 1000, 1500, 2000, 2500]$ and observed qualitatively consistent rankings of method performance (Supplementary Figure 1).

Evaluation threshold For complex matching, we used a neighborhood affinity threshold $\phi = 0.25$, following common practice in previous protein complex identification studies [1, 2].

2.2 Repeated-run stability analysis

Because PCIPG 2.0 contains neural-network-based components, including multi-omics fusion, residue-level graph aggregation and PPI-level graph autoencoding, we further evaluated whether the reported benchmark performance was sensitive to random initialization and stochastic optimization. We repeated the full PCIPG 2.0 pipeline 20 times with different random seeds while keeping all hyperparameters fixed. For each dataset, we report the mean and standard deviation of Precision, Recall, F-score and Acc.

As shown in Table 4, PCIPG 2.0 exhibited stable performance across repeated runs. The standard deviations of F-score and Acc were small on all five benchmarks, suggesting that the observed improvements are robust to random initialization and stochastic training variation.

Table 4: Repeated-run stability of PCIPG 2.0 across five *Saccharomyces cerevisiae* PPI benchmarks. Results are reported as mean ± standard deviation over 20 independent runs with different random seeds.

Dataset	Precision	Recall	F-score	Acc
Collins	0.8290 ± 0.0054	0.8968 ± 0.0048	0.8616 ± 0.0069	0.6788 ± 0.0061
Krogan-core	0.8598 ± 0.0017	0.8163 ± 0.0062	0.8375 ± 0.0003	0.7371 ± 0.0018
Krogan14k	0.7632 ± 0.0001	0.7168 ± 0.0055	0.7393 ± 0.0088	0.6921 ± 0.0043
DIP	0.6753 ± 0.0009	0.7406 ± 0.0029	0.7064 ± 0.0016	0.6174 ± 0.0070
BioGRID	0.6215 ± 0.0004	0.8117 ± 0.0067	0.7040 ± 0.0042	0.6280 ± 0.0007

2.3 Implementation details

To improve reproducibility, we provide the main implementation details used in the benchmark experiments. PCIPG 2.0 does not perform explicit random negative-edge sampling. After the PPI adjacency matrix is constructed, all non-edge positions are included in the non-edge reconstruction term of the loss function.

The model was optimized using Adam with a learning rate of 0.001 and weight decay of 0.01. A StepLR scheduler was used with step size 100 and decay factor $\gamma = 0.8$. The maximum number of training epochs was 2000. The training loss was printed every 25 epochs, and a checkpoint was saved at each epoch. No early stopping was applied. Dropout was used in the GIN component before the final linear projection, with probability $p = 0.5$.

For random seed control, the training script set the seed to 42 and fixed Python `random`, NumPy, PyTorch and CUDA random seeds. Deterministic cuDNN behavior was also enabled.

All experiments were conducted on a Linux workstation equipped with a Quadro RTX 6000 GPU with 24 GB GPU memory and 125 GiB system memory. The NVIDIA driver version was 535.183.01. The workstation also included a 234 GB system partition and two additional storage partitions of approximately 916 GB each.

PCIPG 2.0 is trained in a fully unsupervised manner. The gold-standard complex is not used by the multi-omics enhancement module, structure preprocessing module, graph autoencoder training procedure or complex extraction step.

The evaluation script performs a coverage check on the reference complexes. Specifically, a gold-standard complex is included in the evaluable reference set only when all of its member proteins are present in the model's available protein set. This avoids evaluating the method against reference complexes that cannot be recovered because some of their subunits are outside the model input or lack required structural features.

2.4 Compared methods

To evaluate the performance of PCIPG2.0, we compared it with six state-of-the-art protein complex identification methods covering distinct methodological paradigms, including overlapping clustering, topology-based prediction,

graph representation learning, supervised graph modeling and sequence–interaction integration. All methods were tested on the same five yeast PPI benchmarks, and all predicted complexes were evaluated using the same post-processing, matching and scoring criteria to ensure a fair comparison.

ClusterONE [3]: ClusterONE is a classical overlapping clustering method for protein complex identification. It detects densely connected and potentially overlapping subnetworks in PPI graphs, allowing proteins to participate in multiple complexes.

PC2P [4]: PC2P is a parameter-free topology-based method for protein complex prediction. It identifies complexes from the structural organization of the PPI network without requiring extensive parameter tuning.

PCGAN [5]: PCGAN is a supervised framework for protein complex identification based on adversarial learning. It learns discriminative patterns from known complexes and uses them to improve candidate complex prediction.

AdaPPI [1]: AdaPPI is an adaptive graph convolutional approach for identifying functional protein modules from PPI networks. It leverages graph representation learning to capture informative local interaction patterns.

HGC (HyperGraphComplex) [2]: HGC is a recent method that integrates protein sequence information with PPI data through hypergraph learning, enabling the modeling of higher-order relations beyond pairwise interactions.

SPLP [6]: SPLP was included as an additional representative baseline to broaden methodological coverage in our comparison. It was evaluated under the same benchmark setting and unified scoring pipeline as the other competing methods.

For all methods, predicted complexes were matched to gold-standard complexes using the same neighborhood affinity criterion, and performance was quantified by Precision, Recall, F-score and Acc under a unified evaluation pipeline.

3 Code usability and reproducibility

To clarify the scope of the released implementation, we reorganized the PCIPG 2.0 code and documentation. The revised GitHub repository contains the executable code and a detailed README describing both the original workflow and the enhanced workflow with multi-omics fusion. The complete reproducibility package is archived on Zenodo, whereas the GitHub repository provides the code version and usage instructions.

The released implementation includes both the multi-omics enhancement module and the core structure-aware complex inference module. In the enhanced workflow, the multi-omics module first integrates omics features to generate an enhanced PPI network, and the main PCIPG 2.0 pipeline then uses this enhanced network for structure-aware complex inference. The repository provides scripts for multi-omics feature integration and interactome enhancement, residue-graph construction from protein structures, ProteinMPNN-based residue embedding extraction, structure-aware protein representation learning, PPI-level graph autoencoder training, complex extraction, redundancy removal, benchmark evaluation and sensitivity/ablation analysis.

For usability, the repository includes a one-command entry point for the enhanced workflow. For example, the Collins dataset can be processed using:

```
bash run_all.sh collins
```

This command runs the multi-omics fusion module, saves the enhanced PPI network, copies it to the main pipeline directory and then runs the core PCIPG 2.0 prediction pipeline. The README further explains how to run the original pipeline without multi-omics enhancement, how to replace input files for another dataset and how to update dataset paths when switching benchmarks.

Raw external resources that are subject to the terms of the original data providers are not redistributed unless permitted. Instead, we provide processed inputs, configuration files and instructions for obtaining and preprocessing the corresponding public PPI, omics and structural data sources. The archived Zenodo release corresponds to the exact version used for the revised manuscript, while the GitHub repository will be maintained for future updates.

References

[1] Hongwei Chen, Yunpeng Cai, Chaojie Ji, Gurudeeban Selvaraj, Dongqing Wei, and Hongyan Wu. Adappi: identification of novel protein functional modules via adaptive graph convolution networks in a protein–protein interaction network. *Briefings in Bioinformatics*, 24(1):bbac523, 2023.

[2] Simin Xia, Dianke Li, Xinru Deng, Zhongyang Liu, Huaqing Zhu, Yuan Liu, and Dong Li. Integration of protein sequence and protein–protein interaction data by hypergraph learning to identify novel protein complexes. *Briefings in Bioinformatics*, 25(4), 2024.

[3] Tamás Nepusz, Haiyuan Yu, and Alberto Paccanaro. Detecting overlapping protein complexes in protein-protein interaction networks. *Nature methods*, 9(5):471–472, 2012.

[4] Sara Omranian, Angela Angeleska, and Zoran Nikoloski. Pc2p: parameter-free network-based prediction of protein complexes. *Bioinformatics*, 37(1):73–81, 2021.

[5] Yuliang Pan, Yang Wang, Jihong Guan, and Shuigeng Zhou. Pcgan: a generative approach for protein complex identification from protein interaction networks. *Bioinformatics*, 39(8):btad473, 2023.

[6] Jimit Majmudar and Stephen Vavasis. Provable overlapping community detection in weighted graphs. *Advances in Neural Information Processing Systems*, 33:19028–19038, 2020.
